# The Canadian Landscape of Genetics and Genomics in Nursing: A Policy Document Analysis

**DOI:** 10.1177/08445621231159164

**Published:** 2023-02-27

**Authors:** Rebecca Puddester, Jacqueline Limoges, Sarah Dewell, Joy Maddigan, Lindsay Carlsson, April Pike

**Affiliations:** 1 Faculty of Nursing, 7512 Memorial University of Newfoundland, St. John's, NL, Canada; 2 Faculty of Health Disciplines, 1349 Athabasca University, Athabasca, AB, Canada; 3 School of Nursing, 6727 University of Northern British Columbia, Prince George, BC, Canada; 4 Drug Development Program, Princess Margaret Cancer Centre, Toronto, ON, Canada

**Keywords:** Policy, curriculum policy, innovation and improvement, nursing practice, textual analysis

## Abstract

**Background:**

Genetics and genomics (GG) are transforming approaches to healthcare in Canada and around the globe. Canadian nurses must be prepared to integrate GG in their practice, but modest research in this area suggests that Canadian nurses have limited GG competency. Countries that have integrated GG across nursing provided guidance to nurses about the practice implications of GG through regional nursing policy documents. These documents propelled action to integrate GG across nursing. Little is known about the GG content in the nursing policy document infrastructure in Canada.

**Purpose:**

This study aimed to examine the guidance for GG-informed nursing practice as provided by Canadian nursing organizations in official professional documents.

**Methods:**

Qualitative document analysis was used. A hybrid inductive/deductive analysis approach was used to analyze findings within the diffusion of innovation theory framework.

**Results:**

There is an overall lack of depth and breadth of Canadian nursing documents that include content related to GG. Of the (n  =  37) documents analyzed, four themes were generated including (a) GG guidance in nursing education; (b) regulators’ requirements for foundational GG knowledge, (c) Canadian Nurses Association (CNA) as an early catalyst to GG integration; and (d) early adopters in speciality practice.

**Conclusion:**

There are opportunities to enhance the guidance available to Canadian nurses for the application of GG, through documents of nursing professional associations, nursing education accreditation organizations, and regulatory bodies. Findings suggest oncology and perinatal nurses are the early adopters which is an important consideration in future strategies to implement GG into Canadian nursing.

## Background and purpose

There is a surge of evidence in the scholarly literature highlighting the actual and potential contributions of genetics and genomics (GG) to human health (e.g., contributions to treatment for cancer, mental health, and cardiac conditions (Bilkey et al., 2019; Chen & Meric-Bernstam, 2015; Ngeow & Eng, 2016; Sabour et al., 2017; Stark et al., 2019). Genetics involves the study of specific genes and their roles in disease inheritance while genomics involves the study of all genes (the genome) and how interactions of the genome with the environment affect health and disease susceptibility (National Human Genome Research Institute, 2018). Advancements in genetic and genomic technologies are impacting health care delivery and nursing roles (e.g., GG implications for health promotion and teaching, family health history collection, and interdisciplinary collaboration to ensure continuity of care) (Plavskin et al., 2019). Although nurses in most countries are in the neophyte phase of GG adoption (Tonkin et al., 2020b), many nurse leaders are championing the integration of GG across nursing practice. The Chief Nurse of the International Council of Nurses recently described ‘ten reasons why genomics matters to nursing’ (Acorn, 2022), including the fact that genomics is rapidly moving from specialty practice areas into routine primary care. Nursing organizations and regulatory bodies are striving to provide supportive policies and resources to support the implementation of genomics-informed care. In countries with well-established integration of GG across the continuum of care, nursing organizations have strong policy to support the integration of GG in nursing practice (Kurnat-Thoma et al., 2021; Tonkin et al., 2020a). Policy can be considered “a statement of direction resulting from a decision-making process that applies reason, evidence, and values in public or private settings” (Skelton-Green et al., 2014, p. 88). Nursing policy documents shape professional discourses by establishing boundaries and expectations for nursing knowledge, skills, and judgement and therefore have implications for public protection (Canadian Nurses Association, 2021; College of Nurses of Ontario, 2021). These professional documents can govern, coordinate, and mobilize collective action across institutions and society (Smith & Griffith, 2022). As such, it is imperative to have a foundational knowledge of the GG nursing policy document supports available to Canadian nurses.

### Genetics/genomic nursing policy maturation: The global landscape

The Assessment of Strategic Integration of Genomics across Nursing (ASIGN) tool (Tonkin et al., 2020a) was developed by the Global Genomic Nursing Alliance (G2NA). The ASIGN tool is intended for use by nursing leaders to benchmark the status of GG integration in their countries and/or organizations along a continuum of measurable critical success factors. During the development of the ASIGN criteria, nurse leaders established that policy documents with content relevant to GG, such as competency statements, guidelines and positions statements (Department of Health and Social Care et al., 2022; Kirk et al., 2003; Kurnat-Thoma et al., 2021; National Health Service, 2020; Task and Finish Group, 2011) were critical to the successful integration of GG.

#### Exemplars of successful policy infrastructure

The UK and the US provide exemplars for effective policy guiding the application of GG into nursing practice. The landmark UK government white paper, *Our Inheritance, our future* (Department of Health, 2003) outlined plans to ensure that the National Health Service (NHS) could deliver the benefits of GG technologies to improve the health of UK citizens. This white paper launched several other critical UK policy documents including: the UK genetic competencies for nurses and midwives (Kirk et al., 2003, 2014), a UK parliamentary report recommending genetic curricula standards for nurses (House of the Lords, 2009), and a policy report with recommendations for genomic policy and education in nursing (Task and Finish Group, 2011). These documents generated action for the adoption of GG in nursing. For example, in 2014, £20 million was invested the Genomics Education Program (GEP), a subprogram of Health Education England (HEE) to support workforce development of England's health sector. This educational investment was to ensure that the NHS healthcare workforce (including nurses) was prepared to provide quality genomic health care (Health Education England & Genomics Education Programme, 2017).

These documents also supported the establishment of the UK National Genomic Medicine Service (GMS). The GMS was unveiled in 2019 with seven genomic medicine alliances across the UK, offering mainstreamed genomic testing for specified cancers and rare diseases (HM Government, 2020). Each of the seven genomic medicine alliances had one chief nurse and one senior nurse and/or midwife allocated into its core structure with clearly defined roles (National Health Service, 2020). Even beyond the GMS, nurse-led GG care pathways in the UK are becoming increasingly common. For example, in the familial hypercholesterolaemia (FH) service, patients are referred for specialized nurse assessment for FH risk, with possible genetic testing. If confirmed to have a FH-associated gene, patients are given lifestyle counseling from nurses and further follow-up and treatment as indicated (Wilkinson et al., 2020). Documents such as these can provide some direction for other countries as they develop the infrastructure to integrate GG into nursing practice.

Notable headway with GG integration in nursing has also been observed in the US through the nursing policy document infrastructure (Kurnat-Thoma et al., 2021). GG competency and curricular guidelines exist for both baccalaureate nurses (Consensus Panel on Genetic/Genomic Nursing Competencies, 2009) and advanced practice nurses in the US (Greco et al., 2012). US nurses have also developed policies and recommendations pertaining to GG, e.g., policy recommendations for enhanced federal regulation surrounding direct to consumer testing (Starkweather et al., 2018). The International Society of Nurses in Genetics (ISONG) produced a document interpreting the American Nurses Association code of ethics within the context of GG implications for practice (Tluczek et al., 2019). The US also has resources for nurses to integrate GG into practice, including the *method for introducing a new competency toolkit* (National Human Genomics Research Institute, 2017). These examples demonstrate the powerful impact of policy and documents in providing direction and accountability measures for nursing education and practice.

### Genomic landscape in Canada

While Canada was not included on the list of fourteen countries with actively funded national genomic medicine initiatives (Stark et al., 2019), Canada does have some facilitators for the integration of GG. For example, Genome Canada (https://genomecanada.ca/), a national nonprofit organization exists to promote the Canadian genomics ecosystem. In 2021, the Government of Canada (2022) announced 400 million dollars in funding for a Pan-Canadian genomics strategy to ensure that genomic research findings are diffused through society to benefit Canadians. Additionally, the Canadian Institute of Health Research Institute of Genetics (2022) has a five-year strategic plan entitled, *Sequencing our Future***,** with bold predictions for the integration of genomics in routine healthcare in Canada within the next ten years. Lastly, the Centre of Genomics and Policy at McGill University in Montreal (Centre of Genomics and Policy, 2022) engages in collaboration at the national and international level to conduct translational research examining the ethical, environmental, legal, and social issues surrounding human genomics and policy and to support its implementation to benefit Canadians. While these organizations and their documents are crucial to advancing GG in Canadian society, the nursing profession is never mentioned. As such the implications of these organizations and policies remain unclear to nurses.

A scan of Canadian nursing literature reveals modest research activity to support the application of GG in Canadian nursing practice, although Canadian nursing leaders were highlighting the importance of GG around the same time as the initial activities in the UK and US. For example, Bottorff et al. (2004) reported on a 2001 national planning forum to prepare Canadian nurses for the genomic era of healthcare. At the Canadian forum, a series of recommendations were made (e.g., development of GG nursing educational and practice competencies and the integration of GG into both entry-to practice and continuing nursing education) (Bottorff et al., 2004). Since that time, only four studies were retrieved where Canadian nurses’ competency with GG was measured (Bottorff et al., 2005; Dewell, Seneviratne, et al., 2020; Hébert et al., 2022; Swadas et al., 2022). Participants in the four studies recognized the importance of GG, however, few indicated they could comfortably apply GG in their practice and the majority demonstrated a lack of foundational GG knowledge. Beyond these studies, other than prior research work conducted by members of our team (Dewell, Seneviratne, et al., 2020; Limoges et al., 2022), there is little research evidence that the integration of GG in nursing practice and education is on the radar in Canada. Furthermore, to our knowledge, there is no Canadian research demonstrating the impact or outcomes associated with GG-informed nursing practice.

To accelerate the integration of GG into practice, a pan-Canadian steering committee (www.nursingandgenomics.com) was formed in 2020. This group recognized the importance of GG policy and research and therefore an early endeavor of this group was to examine the Canadian nursing GG landscape. Oriented in the theoretical assumptions of Smith (1974) and her critical concept of a ‘documentary reality’, whereby documents not only reflect but also govern and construct societal reality, the purpose of this paper is to examine content in official Canadian nursing documents that address GG and the extent that they coordinate and mediate the integration of GG into Canadian nursing practice. This is to support the overall aim to inform strategies that support the sustained implementation of GG across Canadian nursing practice*.* This document analysis addressed the research question, “What actual guidance for genetics/genomics (GG)-informed nursing education and practice has been outlined by Canadian nursing organizations in competency, policy documents, and position statements?”

### Theoretical framework: Diffusion of innovation theory

**Figure 1.**
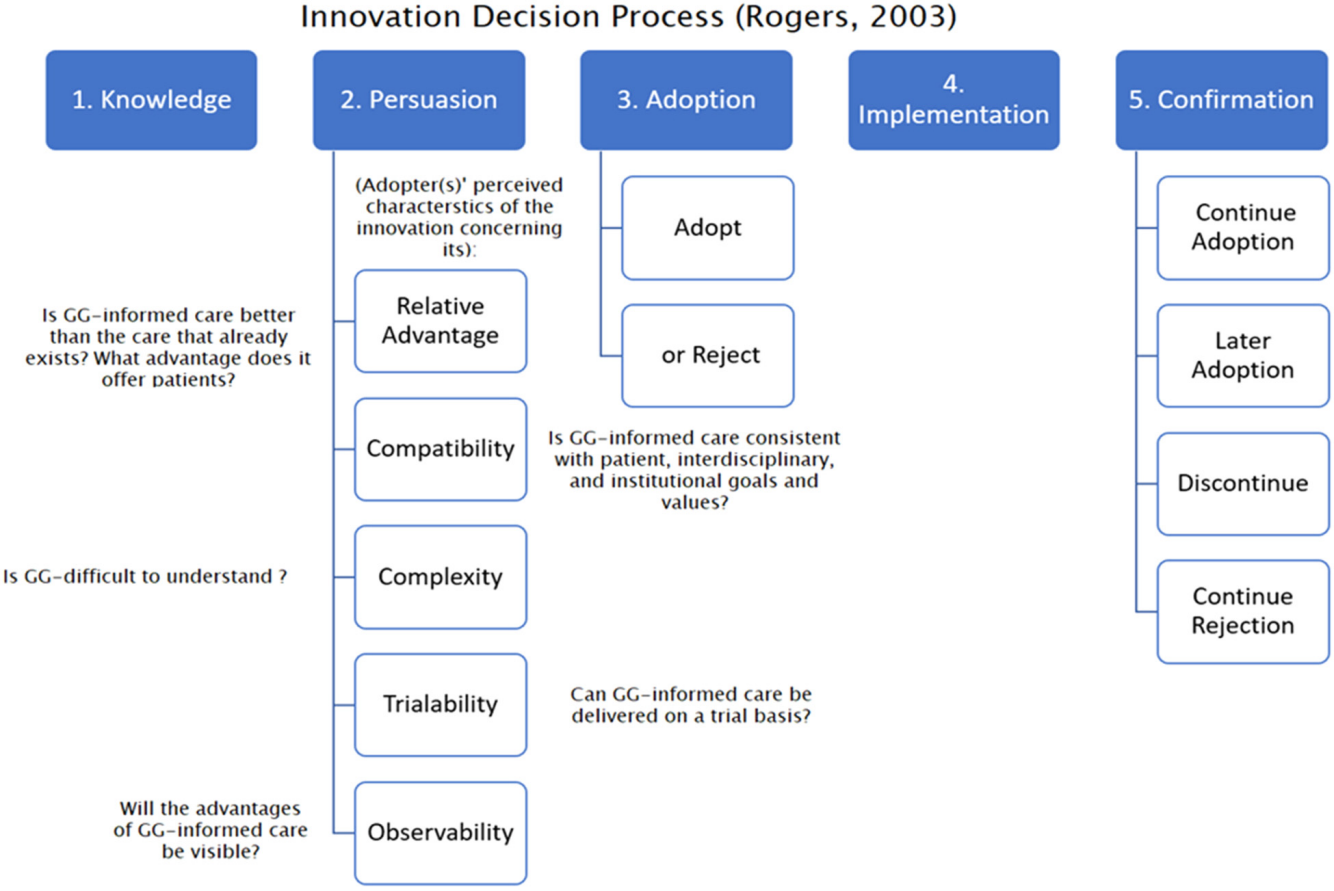
The innovation-decision process (Rogers, 2003).

**Figure 2.**
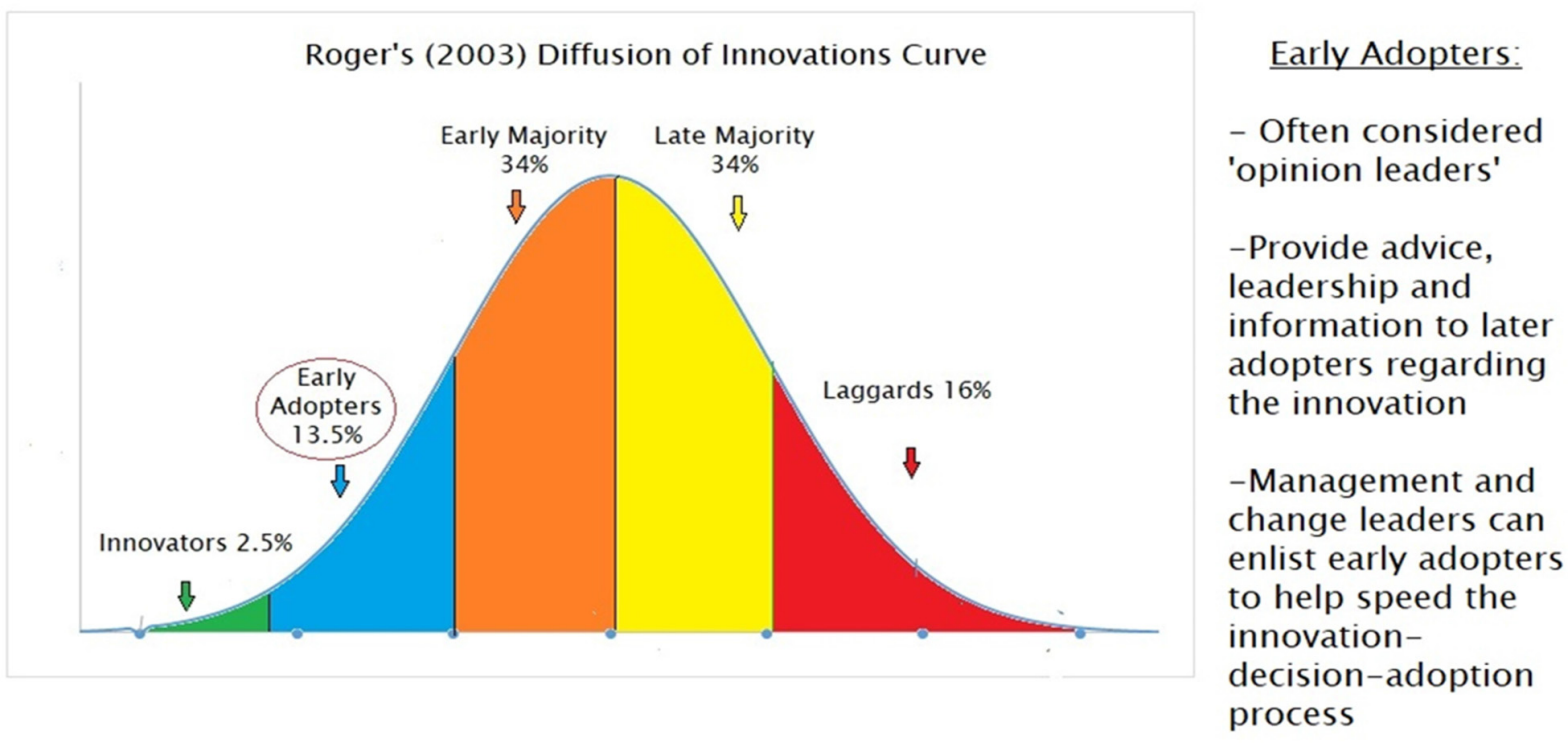
Rogers’ (2003) adopter categories.

The diffusion of innovation theory (DOI) (Rogers, 2003) is a theoretical framework commonly used when examining the decision-adoption process of how innovations, such as GG, are diffused across groups of people and institutions (the adopters). Rogers (2003) considered adoption-decision to be a five-stage process (See Figure 1) and posited that to proceed from the stages of (2) persuasion to (3) adoption, the adopter(s) must hold positive attitudes about the innovation's relative advantage, compatibility, observability, complexity and trialability. Moreover, in Roger's (2003) adoption curve (see Figure 2), he described various categories of adopters by the point in time at which they adopt an innovation (i.e., innovators, early adopters, early majority, late majority, laggards), noting that each adopter group possessed distinguishing characteristics. He highlighted the significance of the ‘early adopters’ cohort as a group that is positioned to accelerate the diffusion of an innovation by influencing others who have yet to adopt the innovation. The DOI has been widely used to explore the integration of GG in nursing (Andrews et al., 2014; Calzone et al., 2012; Dewell et al., 2021; Limoges et al., 2022). The key concepts of the DOI are useful to the exploration and analysis of documents as they permitted interpretation of themes within the context of a relevant implementation theory, as well as to build upon the global GG nursing literature informed by the DOI. As such, the DOI was used as the theoretical framework for this document analysis.

## Methods

Qualitative document analysis (Bowen, 2009) was used to answer the research question. Document analysis is a systematic approach to reviewing and evaluating documents. It is appropriate when the purpose of a study is to elucidate background and context to a phenomenon, highlight areas for further inquiry, provide supplemental data, or to assess change over time (Bowen, 2009). This document analysis was conducted on documents that were retrieved during the period of July 2021 to April 2022. The document analysis was part of a larger research project to develop an engagement framework for nursing and genomics in Canada (Limoges et al., 2022).

A modified version of Fereday and Muir-Cochrane's (2006) inductive-deductive approach to coding and thematic analysis, as endorsed in Bowen's (2009) method, was used to organize the meaningful sections of documents into codes and themes. This approach included five steps: 1. developing an a priori code template based on inclusion criteria and the ASIGN tool; 2. data extraction and initial code reliability; 3. code verification within the context of a theoretical framework; 4. reflexive data analysis and identifying initial themes; and 5. confirmation of themes. Only publicly available and accessible documents were used as data in this study therefore, as per the Tri-Council Policy Statement: Ethical Conduct for Research Involving Humans (TCPS 2) (Canadian Institutes of Health Research et al., 2018), ethical approval was not required.

### Data collection

#### Step 1: Developing inclusion criteria and code template

Inclusion criteria were developed collaboratively with RP, AP, JL and SD. Documents were limited to official Canadian publications written in English post 1990 (coinciding with the beginning of the Human Genome Project). Document selection was guided by the ASIGN tool (Tonkin et al., 2020a). Documents listed under the ASIGN tool as measurable indicators of genomic integration included genomic competency statements, statements from professional organizations that endorse the role and education for nurses with genomics, and codes of ethics that address the ethical implications of genomics for nurses (Tonkin et al., 2020a). Documents of Canadian nursing unions were excluded as these organizations have a mandate to be a collective voice for worker's rights and safe working conditions, which is a different focus than the professional practice implications of GG, the phenomenon of interest in this review. Scholarly journal articles, or documents related to practical nursing education or regulation were excluded. The decision to exclude practical nursing education documents was made following a document scan prior to the full document analysis. The Entry-Level Competencies for Licensed Practical Nurses (Canadian Council for Practical Nurse Regulators, 2019) contained no relevant GG content. As provincial practical nursing guidelines closely mirror national guidelines the decision was made to omit these from the inclusion criteria.

The initial code template was created by RP in consultation with two members of the research team (JL, SD). A template was developed in a Microsoft Word document to sort relevant units of extracted text and to organize them according to nursing organization type (e.g., nursing education accreditors, nursing regulatory bodies, professional associations, specialty practice organizations) to facilitate further data extraction, management and interpretation.

#### Step 2: Data extraction and code reliability

Websites of the official nursing organizations in Canada were scanned by RP for documents that met the inclusion criteria. The following organization websites were searched: Canadian Association of Schools of Nursing (CASN) (the national accreditor for baccalaureate, Masters, and doctoral nursing education) (n  =  1); the websites for the provincial nursing regulatory organizations of Registered Nurses and Nurse Practitioners (n  =  12), registered psychiatric nurses (n  =  5); the Canadian Nurses Association (the national professional association for regulated nurses) (CNA) (n  =  1); as well as the websites of the (n  =  42) member associations of the CNA nursing specialty practice network. From these websites, a total of (n  =  92) documents that met inclusion criteria were skimmed by RP for the presence of the key words “genetic*”, “genome”, and “genomic*.”

Thirty-seven documents were included for analysis (see Table 1). RP skimmed all documents, highlighted significant text and inputted initial codes into a code template. Throughout this process, RP, JL, and SD had frequent discussions to confirm appropriateness of the documents selected and initial codes.

**Table 1.** Total Canadian nursing documents analyzed for GG content.

Document Organization/Type	Organization(s), Year, Location	Description
Canadian Nursing Regulatory Bodies (n = 27)		
Entry-level competencies for Baccalaureate Registered Nurses	BCCNM (2021b) (BC); CCRNR (2019) (National); CRNA (2019) (AB);CRNPEI (2019a) (PEI); CNO (2020) (ON); CRNNL (2019a) (NL); NANB (2019) (NB); NSCN (2020) (NS); RNANTN (2019) (NT &N); (SRNA, 2019) (SK)^a^	1. ELC competency of specialized knowledge: ‘genetics’ listed as part of entry-level RN knowledge base of health sciences 2. ‘Biology and genetic endowment’ listed in ELC as a determinant of health
Registered Psychiatric Nurse Entry-Level Competencies	BCCNM (2022) (BC); CRPNA (2014) (AB); CRPNM (2014) (MN); RPNAS (2014) (SK); RPNRC (2014) (National)	1. ELC of ‘Body of Knowledge and Application’: RPNS must demonstrate knowledge “including genetics and prenatal and genetic influences on development” (RPNRC, 2014) 2. Determinants of health defined as 13 factors including genetics
Nurse Practitioner Entry-Level Competencies	CRNNL (2016) (NL); BCCNM (2021a) (BC); CRNA (2016) (AB); CRNM (2016) (MN); CCRNR (2016) (National); NANB (2016) (NB); NSCN (2016) (NS); RNANTN (2016) (NT&N)	NPs synthesize pharmacologic and non-pharmacologic interventions related to determinants of health (including genetics)
Standards of Practice for Nurses and Nurse Practitioners	CRNNL (2019b) (NL); CRNPEI (2019b) (PEI)^b^; NANB (2019) (NB); YRNA (2013) (YK)	‘Professional Relationships and Leadership’ Practice Standard, the RN/NP advocates on an individual and collective level for healthy public policy informed by determinants of health (including genetics)
National Professional Association (n = 6)		
Position statement of the role of the nurse with reproductive and genetic technologies (RGTs)	CNA (2002a)^c^ (National)	Outlined role of the nurse vis-à-vis RGTs: provide clients information to make informed decisions, referrals, and advocating for availability of information and public engagement in policy surrounding RGTs
Historical overview of RGTs	CNA (2002b) (National)	Factsheet providing a timeline from 1900 to 2001 about key developments in human biology, genetics, assisted human reproduction and cloning
Glossary of terms related to RGTs	CNA (2002c) (National)	Two-page list of main terminologies used in public discussion/debate surrounding RGTs
CNA Code of Ethics for Nurses	CNA (2008)^d^ (National)	** “**genetic endowment” cited as a determinant of health (p. 24) Advances in genetics and genomics described as an important sociopolitical consideration for nursing practice (p. 33)
Canadian Nurse Practitioner Core Competency Framework	CNA (2010) (National)	‘genetics’ listed as a determinant of health (definable entities that are associated with or induce health outcomes) that is incorporated in assessment, diagnosis and therapeutic management of clients and in the evaluation of outcome
Report Proceedings from National Nursing Data Symposium (on CNA document repository)	National Nursing Data Standards et al. (2019) (National)	Outlined future considerations for integrating nursing data with big data (including genomics)
Nursing Education Accreditation Association (n = 1)		
National Canadian Nursing Education Framework (Baccalaureate, Masters, Doctoral)^e^	CASN (2015) (National)	Under *Knowledge* Domain; baccalaureate nursing education programs in Canada will prepare students with “foundational knowledge…from natural & life sciences, and from behavioral & social sciences” (genetics included in this list) (CASN, 2015, p, 8).
Documents from Specialty Nursing Practice Associations (n = 3)		
Perinatal Nursing Standards in Canada	CAPWHN (2018) (National)	1. In pre-conception phase, Perinatal nurse demonstrates knowledge (1.3b) of “genetic risk (e.g., personal and family history of genetic diseases or disorders” (p. 19); 2. In antenatal Phase; (2.4), the perinatal nurse demonstrates understanding indication and implications for prenatal genetic testing
Practice Standards and Competencies for the Specialized Oncology Nurse	CANO/ACIO (2006) (National)	Specialized oncology nurses will a) have an understanding of genetic risk factors, b) apply knowledge of the role of genetics in disease-associated variations to assess genetic family history information, c) provide information to families on the role of genetics in prevention, diagnosis and treatment options, d) assist individuals/families in understanding the processes of genetic counseling and referring them to appropriate genetic information resources and genetic professionals. (p.15)
Radiation Oncology Nursing Practice Standards and Competencies	CANO/ACIO (2015) (National)	Radiation oncology nurses will have an understanding of factors that affect radiation therapy including genetic syndromes

BCCNM: British Columbia College of Nurses and Midwives; CASN: Canadian Association of Schools of Nursing; CCRNR: Canadian Council of Registered Nurse Regulators; CANO: Canadian Association of Nurses in Oncology/Association des infirmières en oncologie; Canadian Association of Perinatal and Women's Health Nurses CRNA: College of Registered Nurses of Alberta; CNA: Canadian Nurses Association; CRNPEI: College of Registered Nurses of Prince Edward Island; CNO: College of Nurses of Ontario; CRNNL: College of Registered Nurses of Newfoundland and Labrador; CRPNA: College of Registered Psychiatric Nurses of Alberta; CRPNM: College of Registered Psychiatric Nurses of Manitoba; NANB: Nurses Association of New Brunswick; NSCN: Nova Scotia College of Nursing; RNANTN: Registered Nurses Association of the Northwest Territories and Nunavut; RPNAS: Registered Psychiatric Nurses Association of Saskatchewan; RPNRC: Registered Psychiatric Nurses Regulators of Canada; SRNA: Saskatchewan Registered Nurses Association; YRNA: Yukon Registered Nurses Association.

^a^ SRNA is now the College of Registered Nurses of Saskatchewan.

^b^ Genetics listed in CRNPEI Standards of Practice for Nurse Practitioners only, not for Registered Nurses.

^c^ This document was retired from the CNA website midway through the research process.

^d^ This document was in the 2008 version but omitted from the most recent (2017) version.

^e^ Genetics is referenced in National Nursing Education framework for Baccalaureate nursing education only, not Masters or Doctoral Nursing Education.

#### Step 3: Code verification within the context of DOI framework

In step 3, initial codes in the template were examined within the DOI theoretical framework (Rogers, 2003) to verify if central concepts of the DOI were applicable in our interpretation of the data as part of our deductive component of the hybrid approach to thematic analysis. For example, we examined if our findings mapped to key postulates in the DOI, such as the key points in the decision-adoption process, and assumptions related to DOI categories of adopters, such as ‘early adopters’ (see Figures 1 and 2).

#### Step 4: Reflexive data analysis and identifying initial themes

In step four, inductive coding was used to generate themes derived from the data and to collapse the codes into categories and themes. Interpretive authority and researcher subjectivity in the generation of themes (Braun & Clarke, 2013; Morgan, 2022) is an important consideration in this type of research. Therefore, the primary coder (RP) made reflexive coding notes about assumptions, values and our pre-existing research and clinical interests in GG. Themes evolved over the course of the analysis as researchers gained further scholarly knowledge of the research phenomenon. Throughout this process, RP and AP met to discuss the data and initial themes.

#### Step 5: Confirmation of themes

In step five, RP discussed themes with two other members of the team with expertise in qualitative research (AP, JM) to come to consensus and ensure rigor of the data analysis. Themes were returned to all members of the research team for reflexive peer debriefing (i.e., exploration of deeper meanings in the data and enhancing the richness of the data through pluralism of researcher perspectives) (Byrne, 2022). This resulted in the development of the final themes.

## Results

Overall, GG content was scarce and there was little evidence of guidance for GG in the 37 documents considered for this analysis. Four themes were generated: (a) GG guidance in nursing education; (b) regulators’ requirements for foundational GG knowledge, (c) CNA as an early catalyst to GG integration; and (d) early adopters in speciality practice.

### GG guidance in nursing education

The national nursing education framework (CASN, 2015) describes the expected outcomes of baccalaureate, masters, and doctoral education in Canada and therefore guides the expected content in Canadian nursing curricula. The framework is organized in six domains (i.e., knowledge; research methodologies, critical inquiry and evidence; nursing practice; communication and collaboration; professionalism and leadership), each of which have guiding principles, and a list of essential components associated with each domain. Under the knowledge domain, genetics is listed as a component of the baccalaureate-prepared nurse's foundational health sciences knowledge across the lifespan (CASN, 2015). There was no reference to the word ‘genomics’ in this document. This brief inclusion of ‘genetics’ in the baccalaureate framework indicates acknowledgement by the national nursing education accreditation body that genetics is relevant to all baccalaureate prepared nurses. However, beyond this, no direction or tools are given related to application of GG in Canadian undergraduate nursing education (i.e., supplemental position statements, competencies, or nursing education directives or resource materials). No mention of GG is made in the national nursing education framework in the context of Masters or Doctoral nursing education.

### Regulatory requirements for foundational GG knowledge

Entry level competency documents (ELC) are important documents developed by the regulators of Canadian nursing practice. They provide guidance about the knowledge and skills that nurses must possess to begin practice as a nurse in Canada. These documents also guide nursing curriculum and provide direction to the public and employers about the requirements of entry-level nurses and what is expected of practicing nurses to maintain ongoing competency (CCRNR, 2019). The word ‘genetics’ is listed in the national CCRNR (2019) ELC for registered nurses as a foundational health sciences knowledge under the competency of *specialized knowledge*. It is grouped with a large list of other health sciences (e.g., anatomy, immunology, psychopathology). This statement is also included in (n  =  10) provincial/territorial registered nurse ELC documents, in the ELC developed by the Registered Psychiatric Nurses Regulators of Canada (RPNRC) (2014) and the four provincial registered psychiatric nurse associations (see Table 1). Under the ELC of *knowledge and application*, it was stated that registered psychiatric nurses should have knowledge of “genetics, and prenatal and genetic influences on development” (p.11). The word ‘genomics’ was absent in all documents retrieved from nursing regulatory associations.

Across the majority of the ELC of regulatory bodies in Canada, genetics/genetic endowment is cited as one of the twelve determinants of health in accordance with the Government of Canada (2019) framework. Under the ELC of ‘*advocacy’* for registered nurses, nurses are expected to advocate for health equity for all by incorporating principles of social justice and the determinants of health (CCNR, 2019). Genetics as a determinant of health is included in the registered nurse ELC documents of (n  =  10) provincial/territorial regulatory bodies. It is also included as a determinant of health in national and provincial registered psychiatric nurse ELC documents (n  =  5), in the nurse practitioner ELC documents in (n  =  8) provinces and territories, and the provincial/territorial standards of practice for Registered Nurses and Nurse Practitioners (n  =  4). It is noted in (n  =  8) Nurse Practitioner (NP) ELC documents that NPs should consider the determinants of health (including genetics) in the assessment and in the selection of pharmacological and non-pharmacological interventions. Under the Standard of ‘*Professional Relationships and Leadership’* in provincial/territorial Standards of Practice document for Registered Nurses and/or Nurse Practitioners (NPs) (n  =  4), it is stated that Registered Nurses (RNs) and NPs have a role to advocate for healthy public policy that takes into consideration the determinants of health (including genetics).

### CNA as an early catalyst to GG integration

Professional nursing associations, such as the CNA, have an important role in “generating the energy, flow of ideas, and proactive work needed to maintain a healthy profession that advocates for the needs of its clients and nurses, and the trust of society” (Matthews, 2012, p.1). Professional organizations often accomplish this through dissemination of policy and procedure documents. Documents from the early 2000's that addressed GG were retrieved from the CNA (national professional association for regulated nurses in Canada) website (CNA, 2002a, 2002b, 2002c). These documents provide evidence of CNA's early acknowledgement of the importance for nurses to have requisite knowledge to integrate GG into practice. For example, to promote the role of nurses with reproductive and genetic technologies (RGTs), the CNA (2002c) released a position statement and noted that “nurses have a crucial role to play in advocating the availability of good information, and public participation in shaping policies about assisted human reproduction; genetic testing; genetic therapy; genetic enhancements; the human genome project; and privacy concerns” (p.1). It was also noted in the position statement that nurses who practice in specialty areas of reproductive health and/or genetic counseling would have important roles in shaping policy and practice surrounding the ethical, legal, and social issues of RGTs. To accompany this position statement, the CNA released two companion documents to ensure that nurses had foundational resources that they could readily retrieve to integrate GG into their practice; a glossary of terms related to RGTs e.g., DNA, genes, genetics, genome, genomics, mutations (CNA, 2002a) and a fact sheet that captured a historical overview of key developments within the field including assisted human reproduction and cloning (CNA, 2002b).

Beyond that period, no CNA documents were retrieved with updates made to the CNA (2002a, 2002b, 2002c) documents despite the considerable advancements in GG sciences over the past twenty years. Nor have any other similar position statements or information sheets been made publicly available. A significant change concerning GG nursing policy content was observed from the previous to current editions of the CNA (2008, 2017) code of ethics for registered nurses. In the 2008 version, “genetic endowment” (p.24) was listed as a determinant of health and “advances in genetics and genomics” (p. 33) was identified as a sociopolitical factor affecting change in the Canadian healthcare system. These statements were absent from the most recent code of ethics for nurses (CNA, 2017).

### Early Canadian nursing adopters in specialty practice

In Canada, 42 member associations are part of a national network of nursing specialities. These specialty groups exist to promote the visibility of speciality areas of nursing and connect nurses who have knowledge and expertise in these speciality areas of practice (Canadian Nurses Association, n.d.). These organizations also disseminate documents to guide specialty nursing practice. There are no recognized specialty or special interest groups for nursing and GG among the Canadian Network of Nursing Specialities. However, “genetic*” and/or “genomic*” was referenced in (n  =  3) documents of speciality standards of practice and competencies. In the Canadian Association of Nurses in Oncology (CANO/ACIO) competencies and practice standards of the specialized oncology nurse (CANO/ACIO, 2006), it was noted that oncology nurses have a role to provide accurate GG education to oncology patients, to counsel patients about genetic risk, and initiate referrals to genetics services where appropriate. In the radiation oncology nursing practice standards (CANO/ACIO, 2015), it was indicated that nurses should include genetic conditions in their nursing assessment as factors that may influence patients’ responses to radiation therapy. In the Canadian Association of Perinatal and Women's Health Nurses (2018) perinatal nursing standards, it was declared that perinatal nurses should be aware of GG considerations during preconception and antenatal care, such as understanding genetic risk factors and indications for prenatal genetic testing.

An interesting finding was the reference made to genomics in a joint position statement on Nursing Informatics released by the CNA & Canadian Nursing Informatics Association (CNIA) (2017). In the statement, it was forecasted that nursing informatics “will continue to evolve, shifting focus in response to emerging technologies and new approaches to care delivery e.g., virtual care, robotics, genomics” (p. 2). Moreover, in a report of proceedings of the National Nursing Data Symposium (National Nursing Data Standards et al., 2019), it was recommended that the Canadian nursing profession needed to be future-oriented in thinking and planning about how nursing data can be integrated with emerging big data sets, such as genomic data.

## Discussion

Analysis of official Canadian nursing documents of the nursing education accreditor (CASN), nursing regulatory bodies, national professional association (CNA), and specialty practice groups reveal a dearth of guidance for integrating GG into nursing education and practice. When GG were noted in these documents, there was little guidance to operationalize the integration of GG to nursing practice and patient care. There was a notable gap between the completeness and range of competency statements in Canada that address GG implications for nursing in comparison to other countries (e.g., the US and UK) as noted in Calzone, Kirk, et al. (2018).

While there was an overt lack and depth of documents retrieved, as noted by Bowen (2009), sparseness or incompleteness of documents can suggest “that certain matters have been given little attention or certain voices have not been heard” (Bowen, 2009, p.33); thus, the sparseness of GG content in Canadian documents retrieved is a significant finding. Based on study findings, we outline areas where guidance provided in policy documents can be optimized. We discuss implications for Canada in the following areas: nursing education accreditation, regulatory bodies, professional nursing associations, and future nursing research. While this discussion focuses on the Canadian context, given that most countries are in the earliest stages of adoption of GG in nursing (Calzone, Kirk, et al., 2018; Tonkin et al., 2020b), these implications likely hold relevance internationally.

### Education accreditation organizations

Overall, there is limited guidance for GG nursing curricula from the national nursing education accreditor (CASN) in Canada and the word ‘genomics’ is currently not included in the CASN (2015) framework. Considering the lack of policy and guidelines outlining competency and curriculum requirements for nurses, prior research findings of limited GG competency in Canadian nursing students are unsurprising (Dewell, Seneviratne, et al., 2020; Swadas et al., 2022). The complete lack of GG content in the CASN (2015) framework at the Masters or Doctoral level is significant given that these cohorts of nurses are traditionally tasked with generating nursing knowledge through research and that these graduates often assume educator roles. Conversely, the US, UK and Japan have educational GG competency statements for nurses (Calzone, Kirk, et al., 2018). Using the US as an example of advanced GG maturity, there are core competencies and curricula guidelines for GG for both baccalaureate-prepared (Consensus Panel on Genetic/Genomic Nursing Competencies, 2009) and graduate prepared nurses (Greco et al., 2012). The baccalaureate core competencies have been endorsed by 47 US-representative nursing organizations and the National Coalition of Health Professional Educators in Genetics (NCHPEG) (Consensus Panel of Genetic/Genomic Nursing Competencies, 2009). Following release of these competencies in 2010, the American Association of Colleges of Nursing mandated GG education into baccalaureate curriculum (Calzone, Jenkins, et al., 2018). As Canada does not have comparable nursing GG competencies, there is little support nor onus on Canadian nursing schools to include GG content in their curricula.

A foundational concept in Canadian nursing is to account for the influence and intersection of the determinants of health (including genetics and genetic endowment). Accounting for the determinants of health assists in upholding social justice and health equity. Without addressing the interaction between GG and other social determinants of health in Canadian nursing education (Dewell et al., 2021), nurses may not be equipped to adequately address equity and social justice. Policy and documented resources can guide the education of future nurses to foster the highest quality holistic nursing care and to fulfill their important patient advocacy roles in the genomic era (Dewell, Benzies, et al., 2020). To address this, the G2NA is in the process of developing minimum global genomic competencies for nurses (Calzone, Kirk, et al., 2018). Although these global competencies are in the earliest stages of development, when finalized, CASN and other nursing educational accreditors can use these to guide the development of Canadian GG competencies.

### Regulatory bodies

Consistent with the findings related to nursing education accreditation, ‘genetics’ is briefly cited as foundational knowledge in the baccalaureate nurse ELC (CCRNR, 2019). However, there is no use of the term ‘genomics’ which means that the implications of clinical whole genome sequencing and/or interactions between the genome and the environment are not officially required knowledge for nursing practice. However, as genomic sequencing is described as one of five key trends that will define the future of health care (Braithwaite et al., 2018), the ELC of ‘*scholar*’ where nurses are expected to keep abreast of “global health care issues and trends to optimize client health outcomes” (p.9) can be leveraged. In keeping with the surge of evidence and scientific advancements in genomics, it is anticipated that Canadian nursing regulators, having demonstrated responsiveness to emerging trends in healthcare previously, will make modifications to the ELC to reflect the requirements of genomic literacy and genomics-informed practice. We anticipate that genomics will follow a similar trend to what has already occurred for medical assistance in dying or nursing knowledge for climate change (CCRNR, 2019). Moving forward, we propose that ‘genomics’ should be included in the ELC as part of foundational knowledge for all nurses. This will help ensure patients can benefit from scientific advances in GG across the continuum of care.

Clarifying GG implications for practice in nursing ELC is possible with pragmatic examples. For example, adding pharmacogenomic implications to the existing ELC expectations for nurses related to pharmacology principles could ensure safest possible medication administration. Pharmacogenomics can reduce mortality and morbidity associated with adverse drug reactions by tailoring a drug to an individual's genetic/genomic make-up (Hippman & Nislow, 2019). Therefore, knowledge of pharmacogenomics can lead to positive patient outcomes (Cheek et al., 2015; White et al., 2019). Adding pragmatic examples aligns with Rogers’ (2003) theory where the adopters’ attitudes towards an innovation, particularly related to its relative advantage and observability, can lead to the decision to adopt an innovation.

‘Genetics’ (not genomics) is widely cited as a determinant of health in ELC and Standards of Practice documents of Canadian nursing regulatory bodies. For example, under the Registered Nurse ELC of ‘Advocacy’, nurses must incorporate knowledge of the “determinants of health, primary health care, and health promotion to achieve health equity” (CCRNR, 2019, p. 8). There are unique ethical, legal and equity considerations inherent to GG-informed healthcare. Underrepresentation of diverse groups in genomic databases and disparity in access to GG testing and targeted treatments threatens to widen existing health disparities if these inequities are not addressed (Atutornu et al., 2022; Gross et al., 2022). If nurses enter practice with the clear understanding and direction for their responsibilities in advocating for equitable GG-informed care, they will be prepared to minimize the harms associated with inequity in GG. Nurses must recognize that human genetic/genomic diversity is largely independent of ‘race’ (Cooper et al., 2003; Lewontin, 1972) to avoid misattributing ‘race’ as a biological/genetic determinant of health disparities. Failure to do so may result in overlooking and potentially reinforcing health disparities of individuals who are negatively impacted by racism (Dordunoo et al., 2022). A statement in ELC and standards of practice that captures the advocacy, ethical, and equity considerations inherent to GG will help support its safe, equitable, ethical translation into nursing practice.

### Professional associations

There were four publications from the CNA following the initial early 2000's movement in nursing and GG (CNA 2002a, 2002b, 2002c, 2008). These publications coincided with the period when Health Canada developed a working group on RGTs and the federal government introduced legislature concerning ethical and legal considerations in genetic and reproductive technologies (CNA, 2002c). Following this period, the momentum to engage with GG dwindled. The reasons for this are unclear, however, it is possible that competing priorities from emerging healthcare trends and issues (e.g., primary health care reform, harm reduction, and climate change) may have displaced GG as a priority for professional nursing associations. Of note in our findings was the lack of GG in the most recent CNA (2017) Code of Ethics for nurses, despite it being included in the prior edition. This discrepancy reflects a disjuncture from documents by the global nursing community. For example, the updated International Council of Nurses (ICN) (2021) code of ethics outlines nurses’ ethical responsibilities related to GG. Moving forward, updating the CNA code of ethics to reflect the ethical and equity issues associated with GG will provide relevant guidance to nurses and support alignment with the ICN (2021) code of ethics for nurses. We also suggest that professional nursing organizations (in Canada and around the world) create position statements to encourage nurses to use GG knowledge in practices that promote health for all.

While it is generally recognized that the UK and US are leading in the integration of GG in nursing, professional nursing organizations in other countries have also created documents to guide nurses’ practice with GG. For example, the Australian College of Nurses (2020) released a position statement, developed in partnership with the G2NA, to set expectations and guide GG-informed nursing action. This position statement has seven key recommendations in both generalized and specialty practice areas. With the recent federal funding for the integration of GG and the mainstreaming of genome sequencing in routine care (Canadian Institute of Health Research Institute of Genetics, 2022; Government of Canada, 2022; Patch & Middleton, 2019), an updated CNA nursing position statement is required to clarify nurses’ contributions and responsibilities associated with these anticipated changes. While these position statements must be contextualized to the Canadian context, we can learn from what has been developed in other countries.

### Informing a plan to move (and keep going) forward

While adding GG content to nursing policy documents is an important step to advance the GG implementation agenda, our findings (i.e., the removal of GG content from the most recent CNA code of ethics) suggest that these approaches alone do not result in sustained change. While GG integration is a complex challenge, the unassailable reality is that the genomics era of health continues to forge ahead; nurses “can accept the changes brought to healthcare by genomics as passive agents or they can be proactive in shaping and informing the transformation in their areas of practice” (UK Task and Finish Group, 2011, p. 11). As a profession, we are presented with an exciting opportunity to stand at the forefront of the clinical translation of GG into safe, ethical, person-centered care. However, the sustained adoption of GG across nursing requires the necessary infrastructure as well as a robust strategic integration plan informed by implementation science.

To this end, the G2NA has rigorously developed excellent resources for the global nursing community to support GG integration across nursing, namely the ASIGN tool (Tonkin et al., 2020a) and the companion road map for global acceleration of genomics integration across nursing (Tonkin et al., 2020b). The roadmap was developed with the Consolidation Framework for Implementation Research (CFIR) (Damschroder et al., 2022), which is based on 19 implementation theories including the DOI that informed this study. The roadmap (Tonkin et al., 2020b) follows the CFIR framework for the stages of the implementation process: planning; engaging (including influencers, champions and leaders); executing; and reflecting and evaluating. Both the ASIGN and the roadmap were designed to be used in tandem. Reiteratively, findings of this study report only on ASIGN tool indicators as they related to the documents of professional nursing organizations in Canada. However, the full ASIGN maturity matrix is a far more comprehensive assessment tool, containing six critical success factors (with corresponding measurable key enablers and indicators) known to support the integration of GG (Tonkin et al., 2020a). We maintain that going forward, ASIGN (and the road map) are helpful resources that nurse stakeholders (e.g., nurse policy makers, and nursing organizations) can use to (a) identify important micro, macro, and meso factors in their regional contexts, (b) direct strategic planning and interventions for GG implementation, and (c) to assess impact and change over time.

This document analysis alone is not a fulsome needs assessment for GG implementation into Canadian nursing. However, this study illuminates the need to develop policies, guidelines and infrastructure to ensure nurses are fully supported for the genomics era. This document analysis also adds to the evidence that in Canada, the nursing profession is in the earliest stages of knowledge and persuasion (Rogers, 2003) in the GG decision-adoption process (Dewell, Seneviratne, et al., 2020), see Figure 1. This confirmed the need for further awareness and knowledge translation activities to engage Canadian nurses to see clinical relevance of genomics and support buy-in that will ultimately lead to adoption of GG (Limoges et al., 2022).

Our findings drew our attention to the early nurse adopters of GG in Canada as suggested by the specialty standards of practice documents (e.g., perinatal nurses and oncology nurses). This finding offers optimism for the possibilities of further GG uptake in these practice areas. This is consistent with Rogers’ (2003) concepts of compatibility and observability, as factors that influence likelihood of an innovation adoption and can help explain why these two nursing specialties are early adopters as the earliest observable implementation of GG in a clinical setting has occurred in the areas of oncology and reproductive health. Leadership, policy and research initiatives involving early adopters of GG could promote widespread engagement with GG throughout the profession (Andrews et al., 2014). The CNG is using this finding on early adopters in Canada to direct strategic actions through knowledge mobilization and engagement activities with the Canadian Association of Nurses in Oncology (Carlsson & Limoges, 2022).

Our findings also highlighted how nursing informatics and GG have intersecting zones of relevancy. Nursing informatics initiatives from Canadian nursing organizations (CASN & Canada Health Infoway, 2015; CNA & CNIA, 2017) can be leveraged for collaborations by identifying synergies, intersecting and complimentary interests associated with the integration of genomics into nursing. Avenues for future research collaborations could involve members of the Canadian Nurses Informatics Association and nurses who conduct research using health technologies. Informatics is an important competency to leverage to deliver the benefits of GG as electronic medical records for collecting family history and electronic GG risk assessment tools are increasingly available (Orlando et al., 2020).

### Limitations

There are noted limitations with the use of document analysis as a method (Bowen, 2009), including potential low retrievability (e.g., some older internet sources may have been removed and therefore were omitted from analysis), as well as potentially insufficient detail, as documents are not typically designed for the purpose of addressing a research question. There is also potential for researcher selection bias and the search was conducted in English only, therefore no Canadian websites were retrieved from province of Quebec.

## Conclusion

Findings from this study suggest that there are many opportunities for Canadian nurse leaders to champion, create and enhance professional practice documents to guide the integration of GG. Furthermore, that a passive approach to the acceleration of GG into nursing practice does not result in sustainable change. While our themes and recommendations were grouped according to nursing organization type, it is important to note that the interests and responsibilities of these nursing organizations overlap and intersect. There are possible synergies through collaboration across domains of nursing practice and across nursing organizations that can accelerate the creation of documentary supports for nurses. Strong, united messaging across all nursing organizations, and use of implementation science are important steps to sustainable momentum to catalyze GG-informed practices that benefit patients, families and communities. Addressing the current gaps in GG content in nursing policy documents should be a collaborative priority of nursing education accreditors, nursing practice regulators, and professional nursing groups. Policy capital from nursing organizations will be an important tool as part of a comprehensive, strategic GG integration plan to ensure continuity of care and that the benefits of GG advances reach all people. Nurse champions of GG in other countries in the earliest stages of GG integration are encouraged to conduct similar analyses of their nursing policy document infrastructure, as well as to refer to the G2NA implementation resources available to identify areas for action to catalyze a GG-literate nursing profession.
